# The Beat

**Published:** 2011-06

**Authors:** Erin E. Dooley

## EU Herbal Product Regs Come into Force

On 1 May 2011 new regulations on herbal products came into force in the European Union (EU).^1^ The regulations require that all products sold be assessed by the European Medicine and Healthcare products Regulatory Agency to show they have been manufactured according to strict standards and contain a consistent and clearly marked dose. Each EU member state also is required to establish a registration scheme for manufactured traditional herbal medicines that are suitable for use without medical supervision. Products will only be sanctioned for use for minor medical problems, such as colds, muscular aches and pains, and sleep problems.

**Figure:**
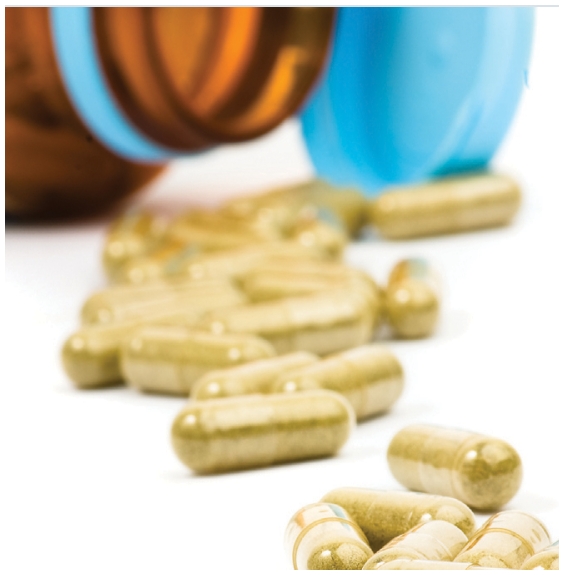
New EU regulations have taken many herbal remedies off store shelves.

## Canadian PM Says No to Ban on Asbestos Exports

In late April 2011 Canadian prime minister Stephen Harper said that country would not ban the export of asbestos, despite calls from scientists and health advocacy groups.^2^ Harper cited concerns about market discrimination in defending the decision and pointed out the chrysotile form of asbestos “is permitted internationally under conditions of safe and controlled use,”^2^ although several studies suggest truly controlled use is impossible to achieve.^3^ Dozens of other countries have banned all forms of asbestos, a known human carcinogen, but the material is still widely used in developing countries, typically with few controls.^3^

## BREATHE LA Launches Child Asthma Management Program in Port City

BREATHE LA, a nonprofit environmental public health advocacy organization, recently renewed its O24u environmental education and asthma management program in the city of Long Beach.^4^ More than 21% of the children in this port city have asthma. The O24u program trains facilitators in 36 Long Beach elementary schools to educate children and their parents about air pollution causes, effects, and asthma identification and management. BREATHE LA estimates the project could yield a 10–15% decrease in school absenteeism due to asthma and a $2.7 million savings in avoided asthma-related emergency room visits and hospitalizations.

## Climate Change: Mastering the Public Health Role

The American Public Health Association (APHA) and the U.S. CDC have released a guidebook for public health practitioners on how to address climate change in their work. The guidebook summarizes a recent series of webinars that brought together climate and health experts and policy makers to discuss climate science, health risk communication, and climate adaptation strategies from a public health perspective.^5^ The guidebook, available for free on the APHA website,^6^ provides examples of how localities are already addressing climate-related health risks to ensure the effects are mitigated for vulnerable populations such as the elderly.

## GreenChill Partnership Now Nationwide

Since 2007 food retailers participating in the U.S. EPA’s GreenChill partnership have voluntarily agreed to adopt refrigeration practices to reduce emissions of refrigerants that damage the ozone layer and contribute to the greenhouse effect.^7^ The agency recently announced the partnership now includes 7,000 partner stores across all 50 states, representing 20% of the supermarket industry.^8^ The U.S. EPA estimates that emissions of refrigerants from partner stores are 50% below the industry average. Extensive best practice guidance at http://www.epa.gov/greenchill/ advises retailers how to safely transition to new refrigerants, reduce the amounts of refrigerants used, and eliminate refrigerant leaks.

**Figure:**
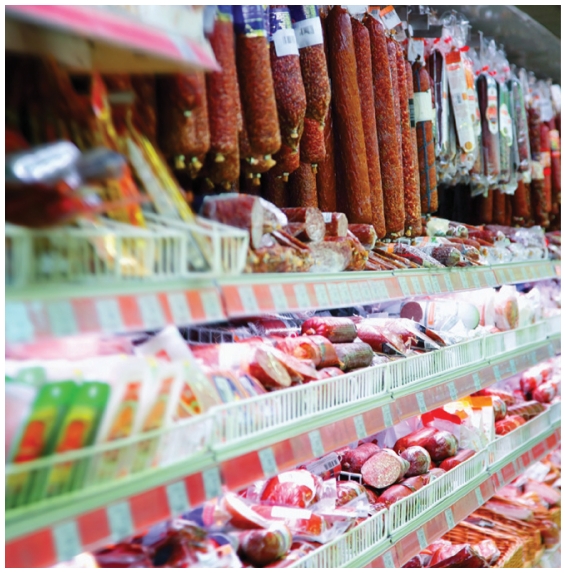
A U.S. EPA initiative gives new meaning to the term “green grocer.”
